# Patterns of peri-gestational weight change among women living with HIV in Nigeria receiving dolutegravir compared to alternative antiretroviral regimens: a retrospective cohort study

**DOI:** 10.1186/s12981-025-00731-x

**Published:** 2025-03-24

**Authors:** Ammar Al Naimi, Charlotte Chang, Holly Rawizza, Oluwaseun Olaifa, Olabanjo Ogunsola, Prosper Okonkwo, Phyllis Kanki

**Affiliations:** 1 Department of Immunology and Infectious Diseases, Harvard T.H. Chan School of Public Health, Boston, MA USA; 2 Department of Obstetrics and Gynecology, Buergerhospital, Nibelungenallee 37-41, 60318 Frankfurt Am Main, Germany; 3 Department of Obstetrics and Perinatal Medicine, Goethe University Hospital of Frankfurt, Frankfurt, Germany; 4 Brigham & Women’s Hospital, Boston, MA USA; 5 APIN Public Health Initiatives, Abuja, Nigeria

**Keywords:** Dolutegravir, DTG, HIV, Gestational weight, Weight change

## Abstract

**Background:**

Weight change for women living with HIV (WLWH) who receive dolutegravir (DTG) is understudied around pregnancy. The aim of this study was to investigate the direction and magnitude of weight change among WLWH pre-, during, and post-gestation based on DTG exposure history.

**Methods:**

This retrospective cohort study evaluated adult pregnant WLWH receiving antenatal care between 2016 and 2022 at two clinics in Nigeria and followed them over three 9-month periods (pregestational, antenatal, and postgestational). Patients were stratified into three DTG exposure groups for each follow-up period: non-DTG, DTG-switch, and DTG. Three mixed effects models with random intercepts and slopes were utilized to assess the association between DTG and weight. Sensitivity analysis was conducted using binomial DTG exposure with starting time.

**Results:**

The study included 2386 women, 851 (35.7%) of whom used DTG at some point. Average maternal weight was 63.8 ± 12.7 kg, 67.0 ± 13.1 kg, and 64.5 ± 12.7 kg during the pregestational, antenatal, and postgestational period. The weight difference in kg for DTG and DTG-switch compared to other ARTs were 0.06 (-1.66, 1.79) and -2.11 (-5.33, 1.11) pregestational, -0.613 (-2.14, 0.92) and 1.21 (-0.80, 3.21) antepartum, and 2.64 (0.37, 4.91) and 0.89 (-1.40, 3.18) postgestational. The antenatal slope (β) for DTG exposure and initiation time was 0.01 (0.001, 0.02) kg/day.

**Conclusions:**

DTG therapy is associated with more rapid weight gain during pregnancy without significantly affecting the total weight gained. Moreover, retained weight postgestation is higher in women on DTG. Therefore, they could face higher future metabolic and cardiovascular risks.

## Background

HIV is a global public health threat with around 37 million people living with HIV (PLWH) worldwide. Efforts to control the epidemic have not met the United Nations Sustainable Development Goals and the global incidence to mortality ratio is as high as 1.94. Sub-Saharan Africa bears the highest burden despite significant progress in HIV control [1]. The prevalence of HIV remains high in Africa such that one in four pregnant women is a woman living with HIV (WLWH) [2].

One of the currently preferred antiretroviral therapy (ART) regimens includes dolutegravir (DTG). DTG is a second-generation integrase strand transfer inhibitor (INSTI) that was approved by the U.S. Food and Drug Administration (FDA) in 2013, and its advantages include convenience, low cost, effectiveness against resistance, and few drug-drug interactions [3]. DTG combined with a dual nucleoside reverse transcriptase inhibitor (NRTI)-backbone is a safe ART regimen during pregnancy, and therefore is recommended for WLWH with reproductive potential [4–6].

The association between clinically significant weight gain and DTG has been shown in several studies and trials. DTG therapy over 48 weeks was associated with 5 kg (kg) weight gain and 12% incidence of obesity. Moreover, the adjusted odds ratio of increasing body weight by 10% after 2 years of DTG was 1.37 compared to other regimens [7–9]. Despite decreased mortality risk associated with an increased body mass index (BMI) after starting ART, this BMI increase might increase the risk of diabetes and other cardiovascular diseases [10]. Moreover, excessive weight gain in pregnancy could be associated with adverse obstetrical outcomes such as preeclampsia, hypertension, gestational diabetes, and premature birth [11–14].

Published data about weight gain and weight changes for WLWH who receive DTG treatment during and after pregnancy are scarce. Total gestational weight gain is the absolute change in weight between the last menstrual period and birth. One study reported 0.05 kg/week increase in weight for pregnant WLWH who newly initiated DTG as compared to efavirenz-based ART regimens. DTG was associated with average weight gain of 0.35 kg/week during pregnancy [15]. Given the known correlation between gestational weight gain and serum progesterone levels [16], the extent of DTG-associated weight gain may be augmented by high levels of progesterone during gestation.

The burden of HIV infection in Nigeria has been declining after reaching a peak prevalence of 5.8% in 2001 [17]. Nevertheless, the estimated number of PLWH in Nigeria is greater than 1.7 million [18]. Since 2004, the APIN PEPFAR program has been a leading provider of HIV treatment, care, and prevention services for PLWH in Nigeria. The APIN-PEPFAR Program provided data from two large antenatal clinics for this research [19].

The aim of this study was to investigate the direction and magnitude of weight change for WLWH pre-, during, and post- gestation depending on their DTG exposure history in our Nigerian study population.

## Methods

Routine clinical care data collected from the Nigerian APIN PEPFAR HIV clinical program between 1st January 2016 and 30th June 2022 was utilized for conducting this retrospective cohort study. Through PEPFAR funding, APIN Public Health Initiatives currently supports 28% of PLWH in Nigeria at 664 sites, including 325 prevention of mother-to-child transmission (PMTCT) sites. This study used deidentified data extracted from electronic clinical records from Jos University Teaching Hospital (JUTH), Plateau State, and Federal Medical Centre Makurdi (FMCM), Benue State, which are tertiary health institutions in north-central Nigeria with over 4000 total annual births. Inclusion criteria were age ≥ 18 years, confirmed HIV-1 status, antenatal care record during the study period, and providing written informed consent for the use of data in secondary research. Exclusion criteria were infection with only HIV-2 and age below 18 years at the start of follow-up. The electronic records of the adult care visits, antenatal care visits, laboratory results, and pharmacy pick-ups over the period of 9 months prior to the last menstrual period and up to 9 months after due date were acquired and linked. Inconsistent records and women with missing gestational age/last menstrual period date or ART exposure information were further excluded from the analysis. Only datapoints with recorded weight and known ART were analyzed; therefore, there is no missing data for the exposure and outcome variables.

The follow-up time was divided into three approximately 9-month periods: pregestational, antenatal, and postgestational. WLWH were classified into three ART exposure groups for each period based on pharmacy records: the DTG group was receiving DTG prior to the start of given period, the DTG-switch group started the period on any different ART and switched to DTG, and the non-DTG group did not receive DTG during the entire course of given period. The descriptive statistics included either mean and standard deviation or median and interquartile range (IQR) for continuous variables, and frequency and proportion for categorical variables.

Three mixed effects models with randomly varying intercepts and slopes were utilized to assess the effect of ART exposure group on the outcome of weight in kg for each of the follow-up periods. Utilizing three separate mixed effects models was deemed appropriate due to the fundamentally different linear weight trends for each of these periods. The models allowed the mean values of the intercept and slope to depend on ART and included the main effect of DTG exposure, a linear time (in months) trend, and ART by linear time trend interaction as fixed effects. The formula of the model can be written as follows:

$${Weight}_{ij}={\beta }_{0}+{\beta }_{1}*{ART}_{ij}+{\beta }_{2}*{month}_{ij}+{\beta }_{3}*{ART}_{ij}*{month}_{ij}+{b}_{0,i}+{b}_{1,i}*{month}_{ij}+{e}_{i,j}$$

Sensitivity analysis was conducted by utilizing binomial DTG exposure, i.e. DTG and DTG-switch were combined into DTG-initiators and compared to other ARTs, and including the time of DTG initiation and its interaction into the mixed effects model of each follow-up period (i.e. pregestational, antenatal, postgestational). This sensitivity analysis increases the precision for detecting DTG association with weight by including the time interaction-variable.

Stata® (ver. 18, Texas, USA) was used to perform all statistical analyses, with p-value of 0.05 as a cut-off threshold for statistical significance and utilizing the 95% confidence intervals. This study was approved by the APIN Institutional Review Board (ref: IRB-SD) and the Institutional Review Board of the Harvard T.H. Chan School of Public Health (ref: IRB23-0687).

## Results

**Fig. 1:**
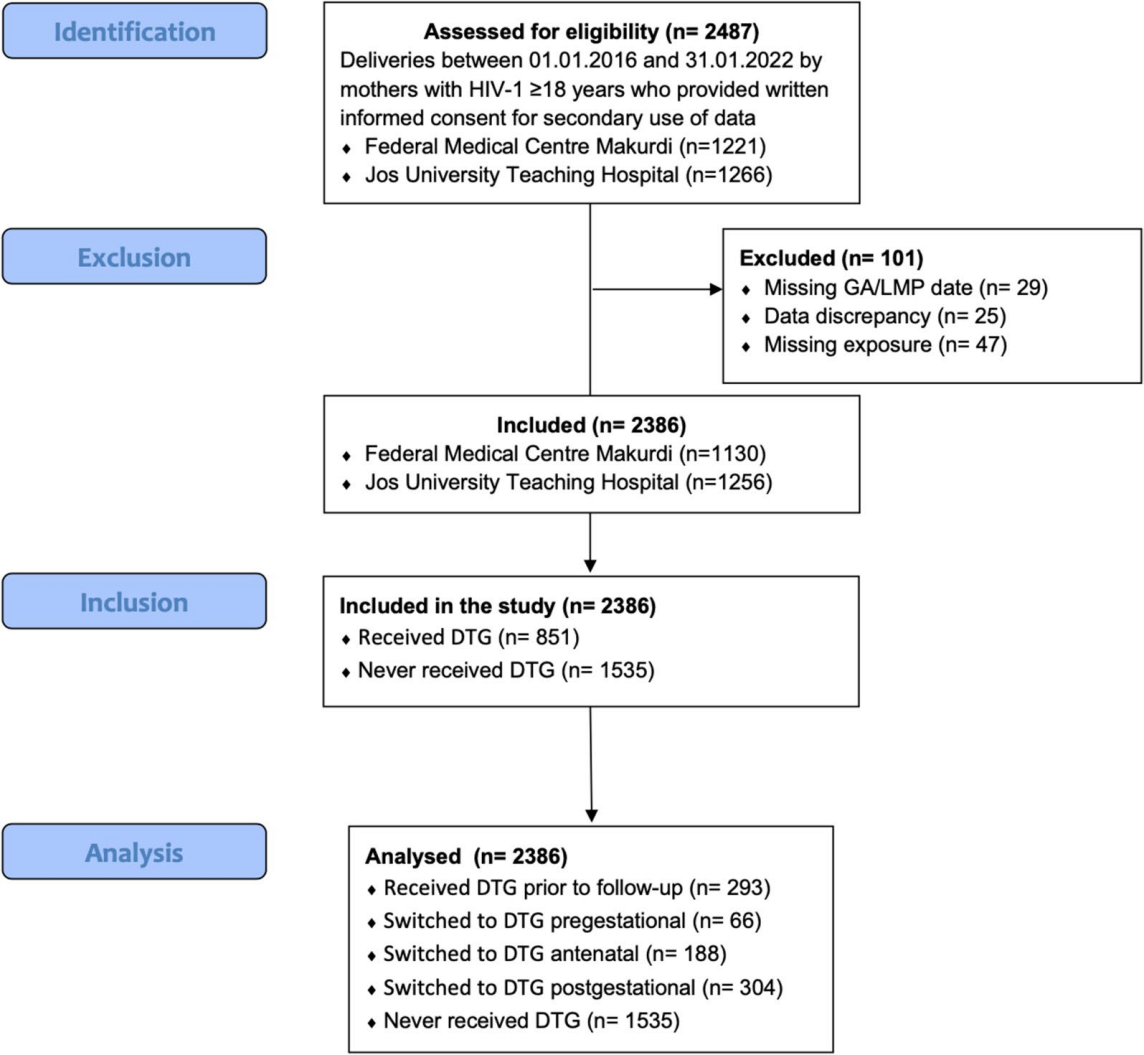
Flowchart of the study cohort

A total of 2386 women were included in this study, of whom 851 (35.7%) received DTG at some point during follow-up while 1535 (64.3%) remained non-DTG users. The number of women who used DTG prior to entering pre-gestational follow-up time was 293. This increased by 66 (mean time to switch: 6.6 ± 1.2 months), 188 (at 4.7 ± 2.8 months), and 304 (at 6.5 ± 2.1 months) women who switched to DTG during the pregestational, antenatal, and postgestational periods, respectively. A study flowchart is shown in Fig. 1.

The follow-up (27 months) for this cohort included 4155, 13,381, and 6367 weight observations in the pregestational, antenatal, and postgestational periods, respectively. Individual participants had a median of 10 (IQR: 6–13) observations. DTG initiators were slightly older (mean 33.6 ± 5 versus 32.7 ± 5 years), more prevalent at FMCM than JUTH (55.7% versus 44.3%), had higher parity, lower baseline RNA viral load, higher CD4 count, and had their first ANC visit slightly earlier (p-values < 0.05) than non-DTG initiators. The demographic characteristics of both cohorts are summarized in Table 1.

**Table 1:** Summary of demographic data by DTG-exposure, with frequency of missing observations for each variable. Data presented as mean (standard deviation), median (IQR) or frequency (proportion, %)

Demographic data (N = 2386)		Non-DTG initiators (N = 1535)	DTG initiators (N = 851)
Maternal age (YEARS)		32.7 (5.0)	33.6 (5.0)
Gravidity		4 (3–4)	4 (3–6)
parity		2 (1–4)	3 (1–4)
History of hypertension (index pregnancy)		19 (1.4%)	6 (0.8%)
History of diabetes (index pregnancy)		4 (0.3%)	1 (0.1%)
Center	JUTH	879 (57.3%)	377 (44.3%)
	FMCM	656 (42.7%)	474 (55.7%)
Education	None	172 (11.5%)	79 (9.5%)
	Primary	336 (22.5%)	161 (19.4%)
	Secondary	599 (40.1%)	367 (44.3%)
	Tertiary	387 (25.9%)	221 (26.7%)
Occupation	Clerical support workers	84 (5.7%)	44 (5.3%)
	Services & sales workers	684 (46.1%)	392 (47.1%)
	Professionals	92 (6.2%)	59 (7.1%)
	Students	203 (13.7%)	119 (14.3%)
	Housewives	227 (15.3%)	131 (15.7%)
	Unemployed	195 (13.1%)	87 (10.5%)
Married		904 (61.4%)	533 (64.3%)
History of tuberculosis		38 (2.6%)	18 (2.2%)
Risk for preterm birth from previous pregnancies		142 (17.4%)	92 (19.8%)
RNA viral load		14,120 (86,329)	6037 (50,798)
CD4 count		512 (226)	543 (245)
Hemoglobin		12.2 (1.7)	12.4 (6.1)
Art regimens	Efavirenz	386 (25.1%)	–
	Nevirapine	378 (24.7%)	–
	Lopinavir/ritonavir	40 (2.6%)	–
	Other	731 (47.6%)	–
First antenatal care visit (gestational weeks)		22.8 (7.4)	21.2 (8.0)
Weight at baseline (kg)		64.0 (13.8)	63.9 (12.5)
BMI at baseline (kg/m^2^)		32.6 (7.2)	25.4 (5.8)

**Fig. 2:**
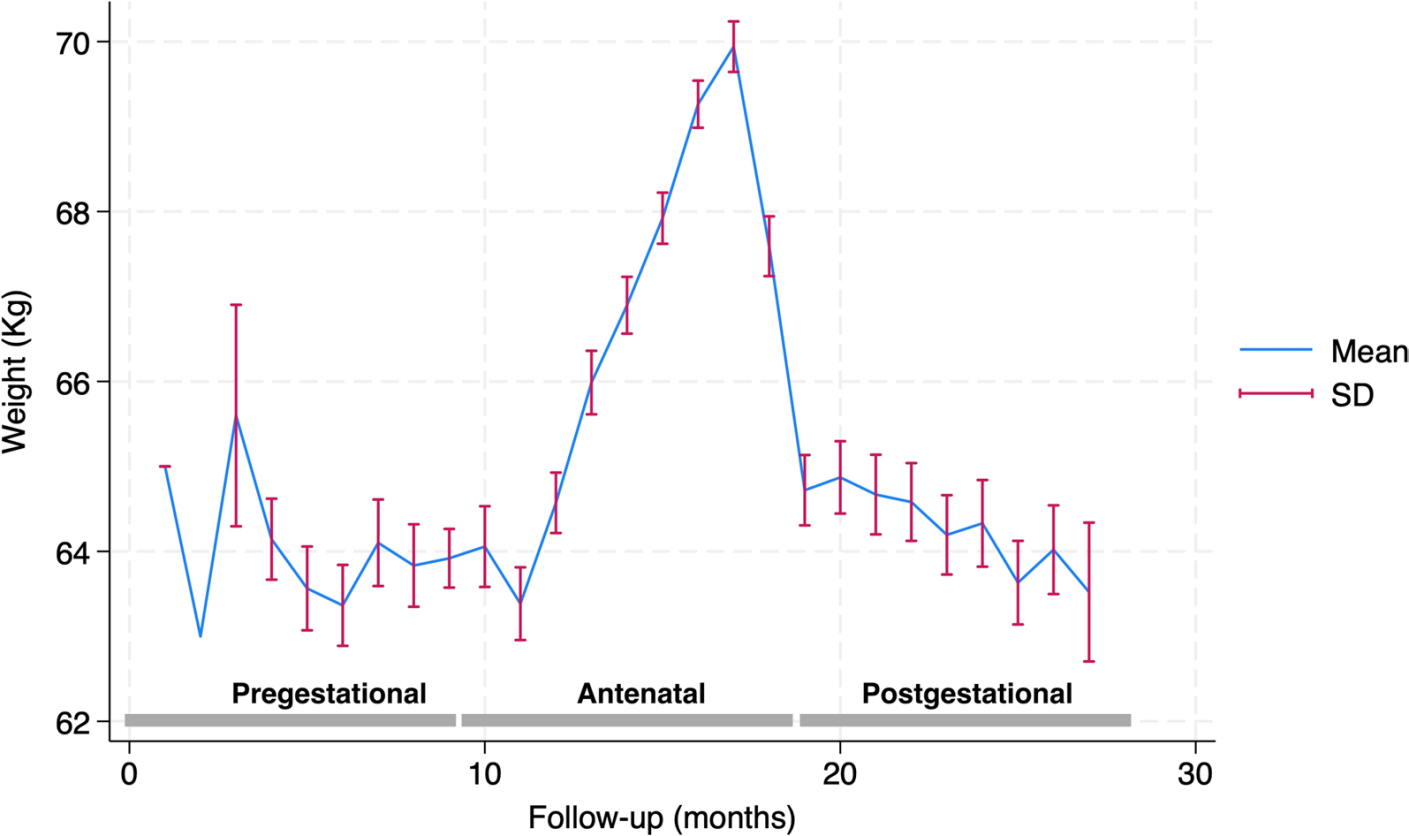
The cohort’s mean weight throughout the entire follow-up time of 27 months. *SD*: standard deviation; *kg*: Kilogram

The average weight per period was 63.8 ± 12.7 kg, 67.0 ± 13.1 kg, and 64.5 ± 12.7 kg during the pregestational, antenatal, and postgestational periods, respectively, and the change of the mean cohort weight over follow-up time is shown in Fig. 2.

The β_1_ coefficients (kg) of DTG and DTG-switch compared to other ARTs were 0.06 (−1.66, 1.79) and −2.11 (−5.33, 1.11) pregestational, −0.613 (−2.14, 0.92) and 1.21 (−0.80, 3.21) antepartum, and 2.64 (0.37, 4.91) and 0.89 (−1.40, 3.18) postgestational respectively. This indicated that despite losing weight up to nine months after delivery, the DTG group remains 2.64 kg heavier on average compared to other ARTs. The components of the three mixed effects models are summarized in Table 2.

**Table 2:** The parameters, coefficients and estimates of three mixed effects models with random slope and random intercept using weight in kilograms as an outcome.

Main mixed effects model (outcome: weight)	PREGESTATIONAL		ANTENATAL		POSTGESTATIONAL	
Parameter	β Coefficient (0.95 CI)	P-value	β Coefficient (0.95 CI)	P-value	β Coefficient (0.95 CI)	P-value
EXPOSURE (reference non-DTG) β_1_						
Switch to DTG	−2.11 (−5.33, 1.11)	0.198	1.21 (−0.80, 3.21)	0.239	0.89 (−1.40, 3.18)	0.448
DTG	0.06 (−1.66, 1.79)	0.941	−0.613 (−2.14, 0.92)	0.433	2.64 (0.37, 4.91)	0.023
TIME (months) β_2_	0.08 (−0.01, 0.17)	0.075	0.71 (0.66, 0.76)	< 0.001	−0.15 (−0.22, −0.09)	< 0.001
Exposure-time-interaction (reference non-DTG) β_3_						
Switch to DTG#Time	0.03 (−0.35, 0.41)	0.868	0.03 (−0.12, 0.18)	0.713	−0.05 (-0.18, 0.09)	0.508
DTG#Time	0.09 (−0.19, 0.36)	0.527	0.07 (−0.05, 0.20)	0.237	−0.11 (−0.26, 0.03)	0.125
INTERCEPT	63.72 (63.02, 64.41)	< 0.001	63.05 (62.42, 63.67)	< 0.001	66.05 (64.98, 67.12)	< 0.001

Random-effects parameters ID: unstructured	Estimate (0.95 CI)	Estimate (0.95 CI)	Estimate (0.95 CI)
Slope	0.93 (0.76, 1.14)	0.36 (0.30, 0.43)	0.32 (0.25, 0.41)
Intercept	156.39 (145.22, 168.41)	160.27 (150.04, 171.19)	184.25 (162.54, 208.86)
covarience (slope,intercept)	2.77 (1.65, 3.90)	−2.55 (−3.20, −1.90)	−3.65 (−4.88, −2.42)
Residual	8.60 (7.96, 9.29)	21.94 (21.31, 22.60)	15.53 (14.77, 16.33)

*CI* confidence interval

**Fig. 3:**
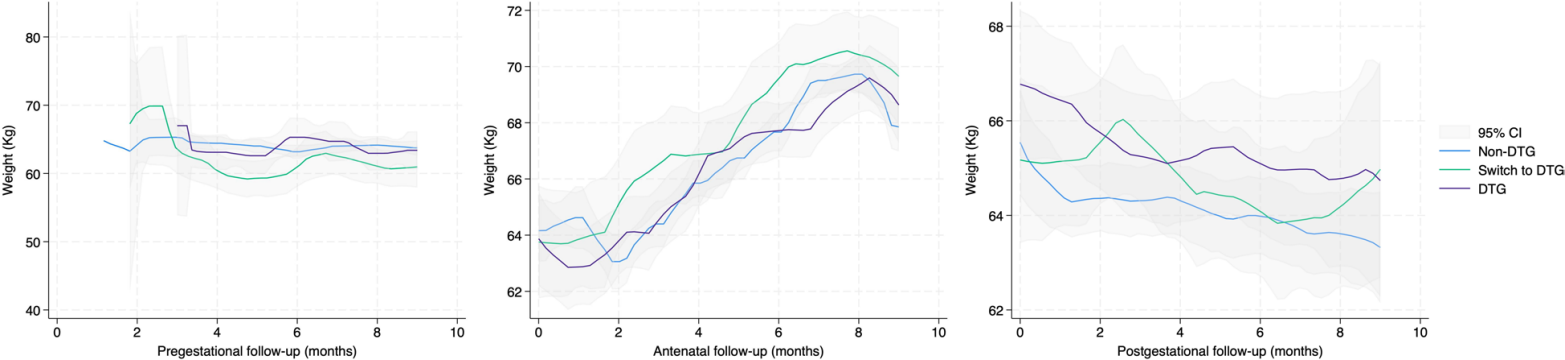
Smoothed trends of weight change over time for three ART exposure groups (Non-DTG, Switch to DTG, and DTG) during the pregestational, antenatal, and postgestational follow-up periods

The trends of weight change over time for the three ART exposure groups in each period, as apparent from smoothed graphs in Fig. 3, indicated no differences in the pregestational period, a more rapid weight gain for the DTG group during gestation and a higher sustained weight for the DTG group postgestation.

In the sensitivity analyses, the mixed effect model using ART exposure as a binomial variable of DTG use, including the time of DTG initiation and its interaction resulted in statistically significant β coefficient of 0.01 (0.001, 0.02) and a p-value of 0.03 for the interaction term during the antenatal period. This indicated an increase in rate of gestational weight change by 0.01 kg for each day of DTG exposure during pregnancy. The components of the three sensitivity analysis mixed effects models are summarized in Table 3.

**Table 3:** The parameters, coefficients and estimates of three mixed effects models with random slope and random intercept using weight in kilograms as an outcome and DTG with initiation time as exposure.

Main mixed effects model (outcome: weight)	Pregestational		Antenatal		Postgestational	
PARAMETER	β Coefficient (0.95 CI)	P-value	β Coefficient (0.95 CI)	P-value	β Coefficient (0.95 CI)	P-value
ART (binomial DTG initiators)	−1.94 (−6.08, 2.21)	0.36	−3.01 (−7.09, 1.06)	0.15	−2.70 (−17.51, 12.10)	0.72
DTG Starting time	−0.0004 (−0.008, 0.007)	0.92	−0.006 (−0.015, 0.002)	0.16	0.005 (−0.07, 0.08)	0.91
ART# DTG starting time	−0.01 (−0.04, 0.01)	0.35	0.01 (0.001, 0.02)	0.03	−0.009 (−0.09, 0.07)	0.81
TIME (months)	0.08 (−0.33, 0.49)	0.71	0.71 (0.40, 1.01)	** < 0.001**	−0.55 (−1.61, 0.52)	0.32
ART#TIME	−0.04 (−0.74, 0.65)	0.91	0.06 (−0.26, 0.37)	0.72	0.22 (−0.85, 1.29)	0.69
DTG starting time # time	0.00002 (−0.001, 0.001)	0.98	0.0001 (−0.0005, 0.0008)	0.70	0.0001 (−0.005, 0.005)	0.97
ART# DTG starting time # Time	−0.001 (−0.006, 0.004)	0.65	−0.0003 (−0.001, 0.0005)	0.47	0.0002 (−0.005, 0.005)	0.94
Intercept	63.88 (61.08, 66.68)	< 0.001	66.19 (62.26, 70.11)	< 0.001	71.78 (57.10, 86.47)	< 0.001

Random-effects parameters ID: unstructured	Estimate (0.95 CI)	Estimate (0.95 CI)	Estimate (0.95 CI)
Slope	0.80 (0.51, 1.24)	0.26 (0. 19, 0.36)	0.11 (0.03, 0.34)
Intercept	141.56 (124.96, 160.36)	152.49 (137.44, 169.20)	140 (109.18, 179.53)
Covarience (slope,intercept)	2.29 (0.33, 4.25)	−1.59 (−2.45, −0.74)	−0.81 (-2.70, 1.09)
Residual	12.24 (10.69, 14.01)	19.23 (18.37, 20.12)	15.66 (14.27, 17.19)

*CI*: confidence interval

## Discussion

This study, to our knowledge, is the first to follow WLWH on DTG prior to, during and after pregnancy and examine their weight change. We opted to utilize three separate mixed effects models because of the contrasting direction of weight change for each observation period. This method of modeling has been previously implemented for differentiating between early and late gestational weight change because of expected difference in trajectory [20]. Our models indicate that women on DTG were, on average, 2.6 kg heavier than those on other ART at the end of follow-up period. This finding supports a previous observation from the DolPHIN-2 study, a randomized controlled trial in South Africa and Uganda in which weight at 72 weeks after birth was around 1 kg higher for women on DTG compared to women on other ARTs [4].

The lack of significant association at p-value > 0.05 between weight and DTG in the pregestational period in our study could be caused by a short follow-up of only 9 months compared to the 72 weeks of postpartum follow-up in the DolPHIN-2 study [4]. Moreover, our small number and proportion of DTG users up to pregnancy of only 293 women could affect the model’s power in detecting an association in the pregestational period. Furthermore, the DolPHIN-2 study was a randomized controlled trial, thus its findings were less susceptible to biases introduced by the retrospective design of our cohort study. ART-naïve people gained weight after starting ART, and WLWH starting DTG-based regimens demonstrated the highest weight gain compared to other ARTs with a mean of 6.1 kg after 18 months [7]. This effect of DTG is not only observed in ART- naïve WLWH, ART-switching to an INSTI leads to weight gain and BMI increase with a magnitude of 5.4 kg/m^2^ within 8 months of a switch [21]. Therefore, each of the follow-up periods would have been too short to demonstrate the change for the switchers taking the median time of switching in consideration. Moreover, the continued lack of association between DTG and weight through the antenatal period could be an effect of a relatively minor additional DTG-associated weight gain in comparison to the physiological gestational weight gain.

One plausible mechanism for the DTG associated weight gain is its proinflammatory effect on neutrophils [22]. A weak association between progesterone levels and weight gain during gestation has been observed [16]; therefore, it can be assumed that higher progesterone levels later in pregnancy could modify the weight change effect of DTG. Women on DTG had a higher gestational weight gain in our cohort of about 4.8 kg, although not statistically significant with p-value of 0.08. While mean gestational weight gain is meaningful, the rate of weight gain is also of clinical importance. Our sensitivity analysis for the mixed effects models revealed a steeper slope for gestational weight gain with DTG. This observation could have a clinically significant impact and should be further investigated, because a steeper slope with upward trajectory of weight gain in the first trimester is associated with gestational diabetes whereas the slope in the second trimester shows no association [11]. Weight gain is a multifaceted process that is significantly affected by behavior, diet, activity and lifestyle factors. The role of these factors in PLWH has not been comprehensively investigated, and a recent meta-analysis from 22 studies showed that diet and physical activity are addressed in only 30% of articles. The absence of concordance concerning the timing of assessment adds more complexity to these important confounders [23]. The lack of information about these confounders in our database is a representation of the challenge in attaining reliable estimates for these factors.

Most of our cohort is classified as overweight and class I obese according to the BMI definitions of the Institute of Medicine. Therefore, their recommended gestational weight gain lies in the range of 4.9–11.3 kg [24]. Our observed gestational weight gain was 4.2 ± 6.8 kg which is lower than the recommendation. Generally, around 70% of women gain gestational weight outside the ranges recommended by the National Academy of Medicine [25]. Moreover, these guidelines were based on a US population without consideration for differences such as stature, race, or ethnicity [26]. Furthermore, gestational weight gain in sub-Saharan Africa is below the recommended ranges and shows variation depending on geography, education, and socioeconomic status. The mean gestational weight gain in Western Africa, which includes Nigeria, is 5.8 ± 0.8 kg [27]. Therefore, our cohort’s gestational weight gain lies within the expected range when taking in consideration that WLWH on ART gain less gestational weight than HIV-uninfected women [15]. Based on our findings, we recommend national surveillance of weight change for WLWH receiving DTG during and around pregnancy with substantial attention to their potentially increased metabolic and cardiovascular risks.

Aside from being the first study to report the peri-gestational weight changes associated with DTG, the strengths of this work lie in a large sample size, lengthy follow-up, high frequency of outcome measure per subject, extensive statistical modelling with sensitivity analysis, and increased generalizability due to use of real-life clinical data. Albeit this work is not without weaknesses. Information bias, missing data, and misclassification are inherent limitation associated with a retrospective design and data linkage. Therefore, adjusted analyses for potential confounders such as socioeconomic status and dietary intake were not conducted either due to completely missing variables or high ratio of missing data. Moreover, patients with missing ART exposure data were excluded from the analysis which could introduce bias. The authors responsible for data accuracy made all attempts to limit the risk of misclassification and linkage problems. Not including the obstetrical outcomes could be considered another limitation, but the obstetrical outcomes were not the aim of this study and were analyzed as a separate research question.

## Conclusions

Our data affirms an association between DTG and positive weight change during and around pregnancy, and pregnancy further accelerates the rate of this weight gain. From a clinically meaningful sample size, our study shows that DTG therapy is associated with a more rapid gestational weight gain. Moreover, post-gestational weight retention is higher in women on DTG, which could still affect metabolic, cardiovascular and future pregnancy outcomes. Therefore, further surveillance of DTG outcomes is essential and will inform national PMTCT policies.

## Data Availability

The dataset used and analysed during the current study is available from the corresponding author on reasonable request.
